# Randomized controlled trial on the effectiveness of absorbable collagen sponge after extraction of impacted mandibular third molar: split-mouth design

**DOI:** 10.1186/s12903-020-1063-3

**Published:** 2020-03-18

**Authors:** Jin-Woo Kim, Tae-Whan Seong, Sura Cho, Sun-Jong Kim

**Affiliations:** Department of Oral and Maxillofacial Surgery, School of Medicine, Ewha Womans University, Seoul, South Korea

**Keywords:** Collagen sponge, Third molar, Surgical extraction, Complication, Periodontal defect, Radiographic

## Abstract

**Background:**

The purpose of this study was to compare the effectiveness of absorbable collagen sponge insertion in tooth extraction sites for socket healing of the impacted mandibular third molar.

**Methods:**

Thirty-six patients with bilateral mandibular impacted third molars based on Pell-Gregory and Winter classification were included in this study. This study was a randomized clinical trial utilizing a split-mouth design with one side assigned as collagen sponge insertion and the other side assigned as the control. Post-operative clinical complications, periodontal integrities, and radiographic outcomes were assessed at 1, 2, and 14-weeks post operatively.

**Results:**

Five patients were excluded during the follow-up period due to loss of follow-up. The study was conducted on 31 patients in total. The mean VAS score of collagen sponge insertion side at 1 week post operation was 1.42 ± 1.26, which was significantly lower than the control side (*P* < 0.05). The mean probing depth of collagen sponge insertion side at 2-week post operation was 5.55 ± 2.28 mm, which was significantly lower than the control side (7.13 ± 1.86; *P* < 0.05). Other various measurements including radiographic outcomes showed no significant group differences.

**Conclusions:**

Placement of collagen sponge after extraction of mandibular impacted third molar reduced early stage post-operative complications and enhanced initial healing of soft tissues and periodontal defects.

**Trial registration:**

This study was retrospectively registered at the WHO ICTRP platform and Clinical Research Information Service, KCT0003363. Registered 21 Sep 2018.

## Background

Extraction of mandibular third molar is a common surgery in oral and maxillofacial surgery. Pericoronitis, dentigerous cysts, periodontal disease, and dental caries in adjacent second molar are common reasons for extraction [1]. In many cases, the mandibular third molar is commonly impacted from unsuccessful eruption due to narrow arch and inadequate space. The extraction of mandibular third molar often leads to complications associated with periodontal defects at the distal surface of the second molar such as bleeding, swelling, pain, trismus, and alveolitis [2, 3].

Various treatment methods have been used to prevent post-operative complications and enhance healing of periodontal defects distal to the second molar. Reported treatment methods include: scaling and root planning the distal aspect of second molar [4], utilizing different flap design for third molar extraction [5–7], varying suturing techniques [8], bone grafting with or without membrane to extraction socket [9], bone grafting with absorbable or non-absorbable membrane, using guided tissue regeneration for new attachment [10], applying autologous platelet rich plasma gel to extraction socket [11], and inserting absorbable collagen sponge to extraction socket.

One of these methods, the placement of absorbable collagen sponge, has been reported to have many clinical advantages. The collagen sponge acts as an extra-cellular matrix, favoring the immigration of osteoblasts, stabilizing blood clots, help soft tissue healing, and aid in wound protection and bone reconstruction [12]. However, there has been no studies assessing the post-operative complications, periodontal defects, and radiographic evaluation of absorbable collagen sponge use in a randomized controlled trial. Thus, the purpose of this study was to investigate the effectiveness of collagen sponge insertion after extraction of impacted mandibular third molar.

## Methods

### Patients and study design

The authors designed a prospective, comparative split-mouth randomized controlled study. Subjects were randomly assigned and divided by split-mouth design into collagen sponge insertion side and control side.

Healthy patients who visited the Department of Oral and Maxillofacial surgery at Ewha Woman’s University Hospital Mok-dong Medical Center for preventive impacted mandibular third molar extraction between July 2013 and June 2015 and satisfying the inclusion criteria were selected for the study. This study was conducted in full accordance with the ethical principles, including the World Medical Association Declaration of Helsinki, and authorized by the Institutional Review Board of Ewha Womans University Medical Center (IRB No. ECT 13-23A-05). Written informed consent was obtained before surgical treatment. This study also adheres to the CONSORT guidelines. The datasets used and/or analyzed for this study is available from the corresponding author upon reasonable request. This RCT is registered at the WHO ICTRP (international clinical trials registry platform) and CRIS (clinical research information service; No. KCT0003363; http://cris.nih.go.kr/cris/en/search/search_result_st01.jsp?seq=12339).

**Fig. 1:**
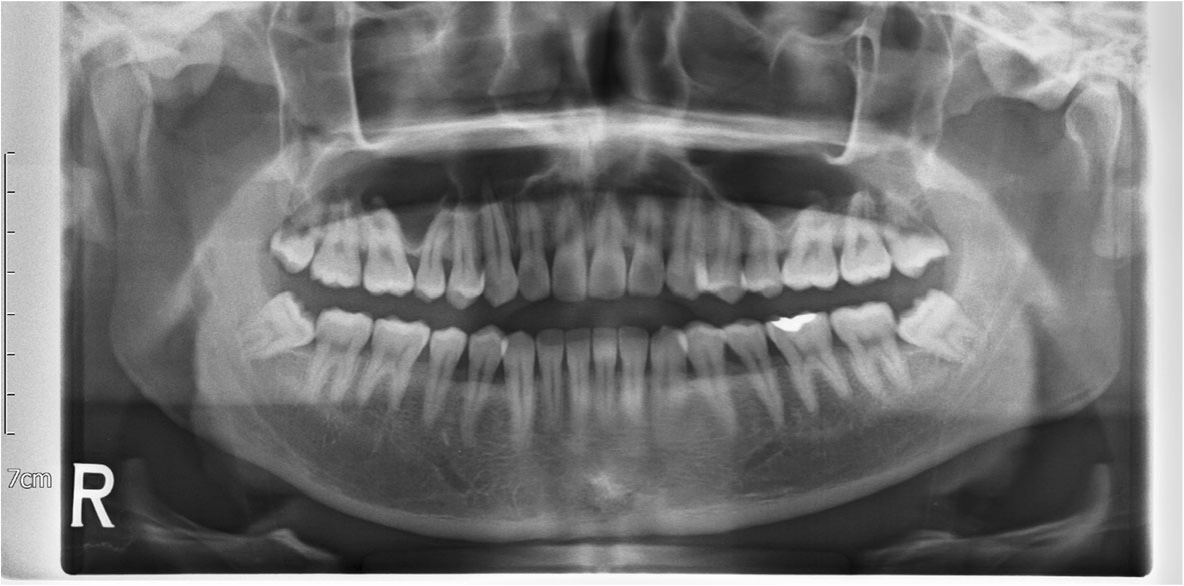
Pre-operative panoramic radiograph showing impacted bilateral mandibular third molars with same Pell-Gregory and Winter classification

Patients with infectious disease (HIV; human immunodeficiency virus, HBV; hepatitis B virus) and patients who had peri-coronitis or peri-apical inflammation were excluded from the study. Patients with bilateral mandibular impacted third molars having same Pell-Gregory (impacted depth and ramus relationship) [13] and Winter classification (impacted angulation) [14] were included in the study. (Fig. 1) Effect size was calculated as 0.728, alpha was 5%, beta was 20%, and the power of test was set at 80%. The sample size required given the specifications and assuming a 10% drop out rate was 34 patients.

The absorbable collagen sponge used in this study was Ateloplug® (Bio-land, Cheong-ju, Chungcheongbuk-do, South Korea). It is composed of 85–95% type I collagen and 5–15% type III collagen derived from porcine skin. Ateloplug® is composed of non-antagonistic atelo-collagen obtained by enzymatic treatment during the manufacturing process to remove inflammation and immune responses. Because atelo-collagen is obtained by removing the telopeptide located at the terminal end of the collagen molecule and uses crosslinking between the amino group and the carboxyl group of the collagen by keeping them at high temperature vacuum state, it has high biocompatibility and excellent cell affinity. Additionally, the porous sponge structure makes its absorbance 15 to 20 times the Ateloplug® weight. Its platelet adhesion test using lactate dehydrogenase enzyme in platelets exhibited hemostatic potential better than that of other collagens. According to the manufacturer, Ateloplug® is completely absorbed within 2 to 4 weeks.

Treatment random assignment for each study participant was done using random number generator from the statistical program SAS (SAS Institute Inc. Cary, NC, USA). Through randomization, patient assignment and right/left assignment were performed in equal numbers. This randomization method determined whether the patient would receive collagen sponge insertion on the right side or the left side. Logically, the remaining side was assigned as the control side. On the day of surgery, both sides of the impacted mandibular third molars were extracted simultaneously. For the experimental group, Ateloplug® was inserted on the test side and for the control group, no insertion was given. Treatment side was masked to both the patient being treated and the research staff, and unmasked to the operator at the time of operation.

### Surgical procedure

Surgical extractions were performed by the same surgeon with the same procedure under intravenous (IV) sedation with midazolam and under local anesthesia using 2% lidocaine containing 1:100,000 epinephrine. Vital sign was monitored and the presence of adverse reactions were checked. After full thickness incision to external oblique ridge and sulcular incision from the distal side of second molar to mid-buccal area of first molar, full thickness muco-periosteal buccal flap was raised and odontomy and ostectomy were performed using low-speed round and fissure bur under constant irrigation with sterile saline. After extraction, the site was sutured for facilitating wound healing with Vicryl® 3–0 and 4–0; Ethicon Inc., Johnson and Johnson, New Brunswick, NJ, USA. Approximately one to three single sutures were used for the envelope flap distal to the second molar, followed by interdental sutures between the first and second molars. In cases where the retention of collagen sponge was considered unstable, crisscross suturing was performed. The participants of this study were patients that satisfied the Pell-Gregory classification and Winter classification and identical suturing technique was used on both sides of each patient. All patients were prescribed antibiotics (Huons Amoxicillin Cap. 500 mg *tid*, Huons, Seoul, Korea), analgesics (Soleton Tab. 80 mg *tid*, CJ healthcare, Seoul, Korea), and antacid (Famotidine Tab. 20 mg *bid*, Nelson, Seoul, Korea) for 7 days following the surgery and were given 0.12% chlorhexidine gluconate mouthwash to use every 12 h for 7 days. Patients visited the clinic at 1 week (for stich out) (T1), 2 weeks (T2), and 14 weeks (T3) post-operatively for follow-up. Panoramic radiographs were taken post-operatively at 1, 2, and 14 weeks.

### Assessment

Primary outcomes of the study were the visual analogue scales (VAS) scores, facial swelling (clinical evaluation), and probing depth (periodontal evaluation). As secondary outcome, lamina dura, overall density, trabecular pattern scoring (radiographic evaluation), and maximum mouth opening were assessed and analyzed.

At first visit, screening and panoramic radiograph was taken and cone beam computed tomography was taken if necessary. Confirmed study participants were screened for pre-operative assessment (T0) including periodontal and radiographic assessment. Post-operative clinical, periodontal, and radiographic assessments were performed at 1 week (T1), 2 weeks (T2), and 14 weeks (T3) post-operatively. The details of the measured parameters are described below.

#### Clinical evaluation

Clinical evaluation was performed based on three aspects: VAS score for pain, facial swelling, and maximum mouth opening. To evaluate the patient’s pain level, VAS score was recorded. VAS score is a visual pain assessment method using numbers ranging from 0 to 10, where 0 indicates ‘no pain’ and 10 indicates ‘very painful’. Facial swelling was evaluated by the method described by Schultze-Mosagu et al. [15] This method measures the distance from the tragus to canthus (oral commissure) and the distance from the tragus to pogonion. The rate of facial swelling was calculated by dividing the preoperative and postoperative difference by the preoperative value and multiplying that by 100 in each of the two base lines. The sum of these two arithmetic values was used to evaluate facial swelling. Maximum mouth opening (MMO) was measured as the maximum distance between the maxillary and mandibular central incisors using a millimeter caliper.

#### Periodontal evaluation

Periodontal defect was evaluated with the following 4 aspects: probing depth (PD), gingival recession (GR), gingival index (GI), and bleeding on probing (BOP). PD is the distance from the gingival margin to the base of the gingival pocket. GR, exposure of the roots from the loss of gingival margin, is the distance from the cemento-enalmel junction to the gingival margin. GI is the gingival inflammation index obtained by probing the gingiva. It is represented by assigning points from 0~3 and the more 0 points there are, the healthier gingiva. BOP is a phenomenon of periodontal tissue bleeding with weak stimuli such as periodontal probing. All of the above periodontal defect evaluations were measured at each of the 4 points of the teeth: mesio-buccal (MB), buccal (B), disto-buccal (DB), and disto-lingual (DL).

#### Radiographic evaluation

Bone healing progression of extraction socket was assessed radiographically using panoramic view. The evaluation of bone healing outcomes and scoring system were based on the methods of Kelly et al. [16] Radiographic reading was evaluated in the following 3 aspects: lamina dura, overall density, and trabecular pattern score. The scoring system set the normal range radiograph as the baseline radiograph and scored it 0. Variation range of scores was + 2 to − 2, and a score of + 1 to − 1 indicated significant changes from the baseline. In evaluating the lamina dura, + 1 indicated thinner and hazier appearance of the lamina dura and − 1 indicated a thicker lamina dura. In evaluating of density, + 1 indicated an increase in radiological density between mild to moderate while − 1 indicated a decrease in radiological density between mild to moderate. In trabecular pattern, + 1 corresponded to a thicker and harder trabecular pattern and − 1 corresponded to a more granular and uniform pattern with no independent glass pole formation.

### Statistical analysis

SAS version 9.4 was used to perform all statistical analyses. To investigate the group difference, Mann-Whitney U test was carried out. To measure the group difference of VAS at each time point, difference-in-difference was estimated. Group difference at T1 was set as the reference. Two surgeons (JW Kim and TW Seong) independently carried out radiographic evaluations and the inter-examiner agreement was calculated as the intraclass correlation coefficient (ICC) from a two-way random model and absolute agreement type. Significance level was set as *P* < 0.05.

## Results

The total number of patients satisfying our inclusion criteria was 36 patients. However, 5 patients were dropped out during follow-up and hence, the study was conducted on 31 patients (15 Male, 16 Female). The mean age of patients was 23 ± 7.14. According to Pell-Gregory classification (impacted depth and ramus relationship) and Winter classification (impacted angulation), impaction depth was classified as level A, B, and C and ramus relationship was classified as class I, II, III. Impacted angulation was classified as mesio-angular, horizontal, disto-angular, and vertical. (Table 1.) Mean operation time was recorded as 28.41 ± 11.34.

**Table 1:** Baseline characteristics of included patients

Variable		N
Patients		31
Age (years)		23.52 (±7.14)
Gender	Male	15
	Female	16
Impaction depth	Level A	12
	Level B	14
	Level C	5
Ramus relationship	Class I	15
	Class II	13
	Class III	3
Angulation	Disto-angular	2
	Horizontal	10
	Mesio-angular	18
	Vertical	1

**Fig. 2:**
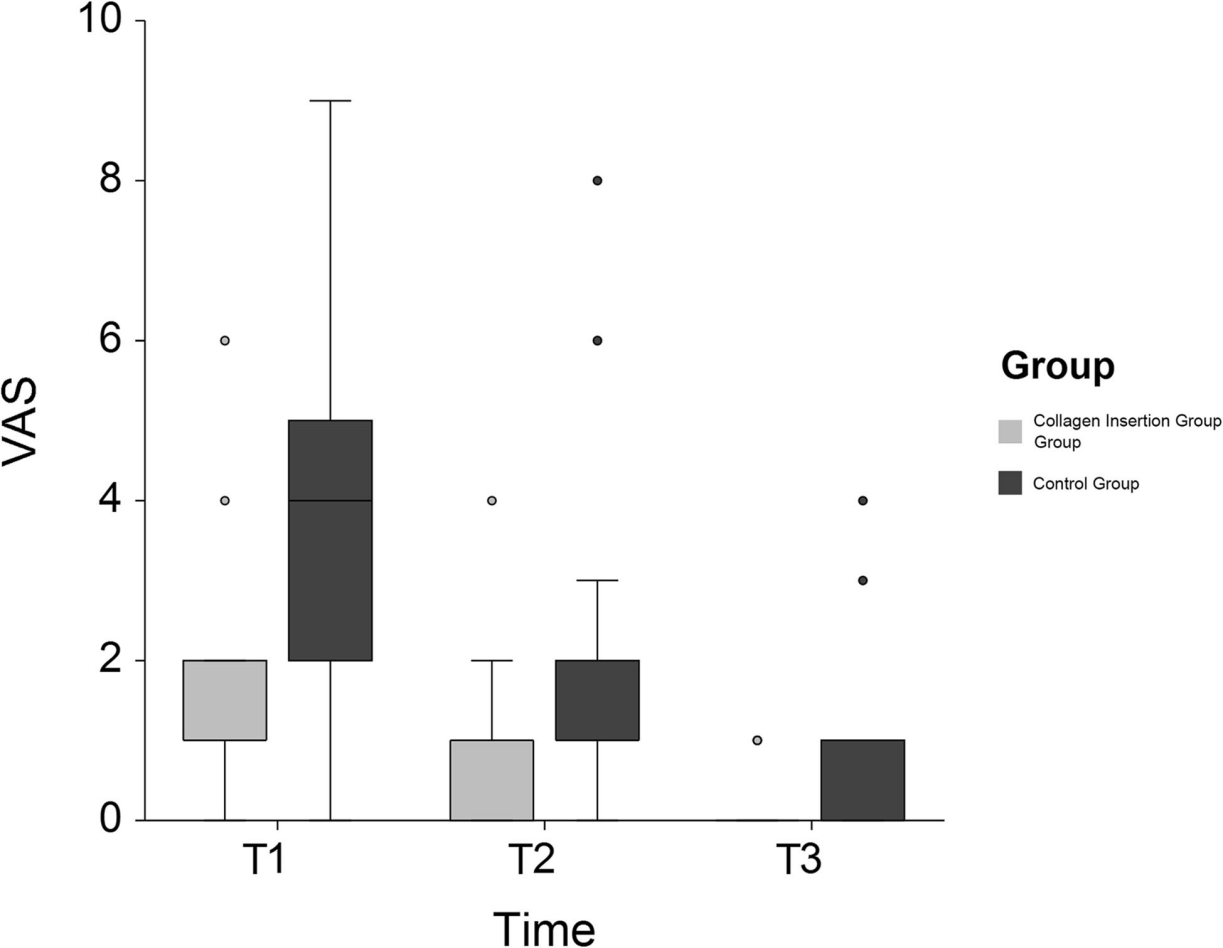
A comparison of the VAS scores for the sides that received a sponge and those that didn’t. The results are shown as the mean (SE). * indicates *P* < 0.05 between collagen sponge insertion versus control. Abbreviations; T1, 1 week post-operatively; T2, 2 weeks post-operatively; T3, 14 weeks post-operatively.

MMO showed a decrease at T1, then gradually increased until T3 recovering to opening close to that of T0. Mean VAS score of the experimental group showed a lower score of 1.42 ± 1.26, whereas control was 3.85 ± 2.43 at T1 (*P* < 0.05; Table 2 and Fig. 2). Difference-in-difference estimates indicated significantly lower score of VAS in the experimental group at T2 and T3 (*P* < 0.01). Facial swelling ratio of collagen sponge insertion side was lower at T1 and T2, but difference between the two groups was not significant. (Table 2.)

**Table 2:** Assessment of clinical complications

Value	Time	Collagen sponge insertion	Control	Difference in difference	Lower 95% CI	Upper 95% CI	*P* value
**VAS score**	*T1*	1.42 (1.26)*	**3.85 (2.43)**	Ref			
	*T2*	0.74 (0.93)*	**1.73 (1.67)**	−1.42	−2.39	−0.45	0.004
	*T3*	0.19 (0.40)	0.42 (0.85)	−2.19	−3.16	−1.23	< 0.001
**Facial swelling (%)**	*T1*	5.76 (11.63)	7.93 (12.28)				
	*T2*	2.09 (8.63)	2.21 (8.79)				
	*T3*	−0.81 (5.98)	−0.91 (6.44)				

Abbreviations; T1, 1 week post-operatively; T2, 2 weeks post-operatively; T3, 14 weeks post-operatively; *CI*, Confidence Intervals

Results are shown as mean (SD)

^*^Indicates significant group difference (*P* < 0.05) between 2 groups

**Fig. 3:**
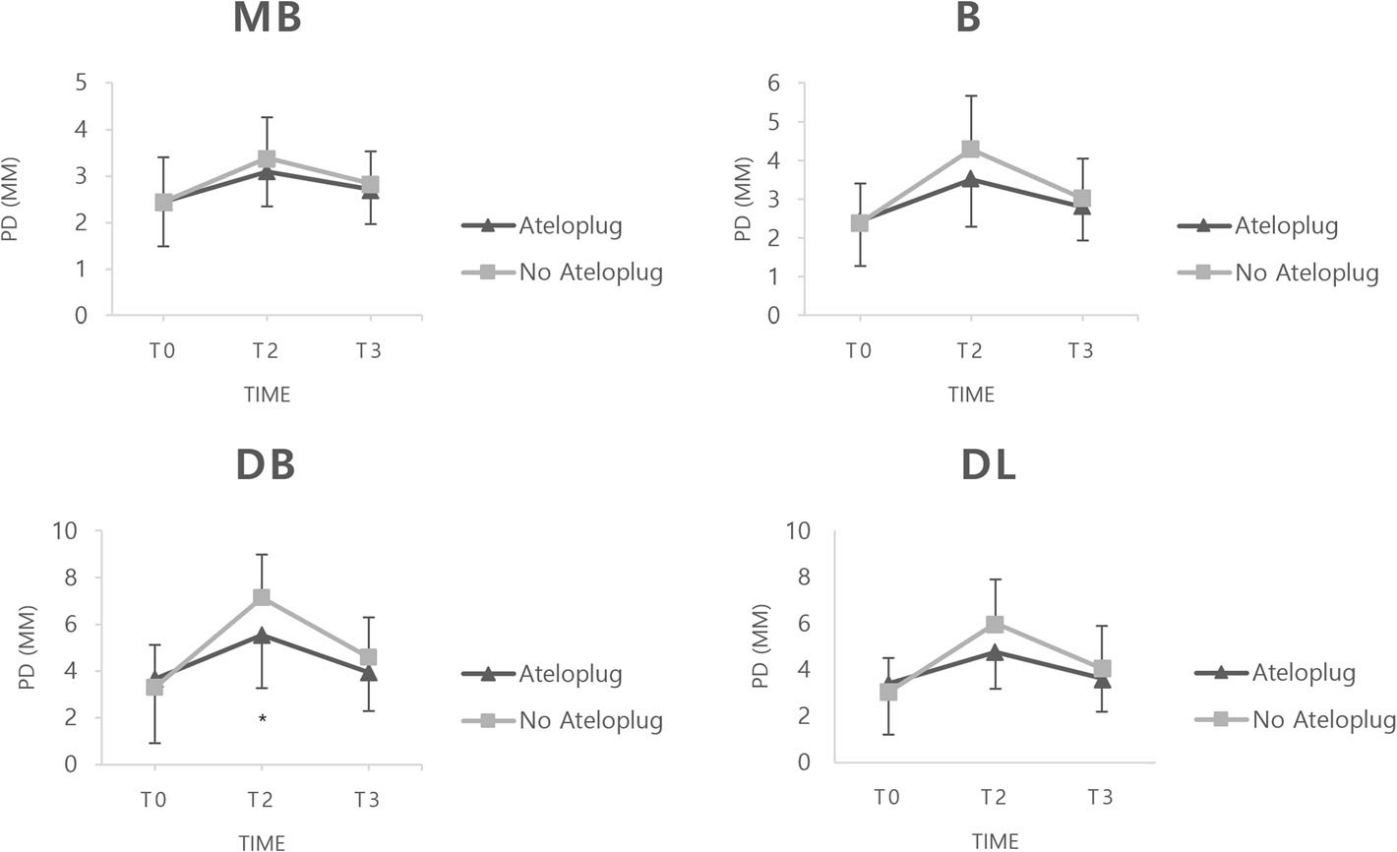
A comparison of the probing depths for the sides that received a sponge and those that didn’t. The results are shown as the mean (SD). * indicates *P* < 0.05 between collagen sponge insertion versus control. Abbreviations; T0, Pre-operation; T2, 2 weeks post-operatively; T3, 14 weeks post-operatively

The mean PD increased after surgical extraction of impacted mandibular third molar and gradually decreased in points MB, B, DB, and DL. The mean PD of collagen sponge insertion side at DB was 5.55 ± 2.28 mm, whereas the control side was 7.13 ± 1.86 mm at T2. (*P* < 0.05; Table 3 and Fig. 3) The mean GI and BOP also increased at T1 and gradually decreased at all points. However, the differences between the two groups were not significant. GR both increased and decreased over time, but the difference between the two groups were not significant. (Supplementary table S1.)

**Table 3:** Assessment of probing depth

Value	Time	Collagen sponge insertion	Control
**Probing depth**			
Mesio-Buccal	*T0*	2.45 (0.96)	2.45 (0.96)
	*T2*	3.10 (0.75)	3.39 (0.88)
	*T3*	2.71 (0.74)	2.84 (0.69)
Buccal	*T0*	2.45 (1.18)	2.39 (1.02)
	*T2*	3.52 (1.23)	4.29 (1.37)
	*T3*	2.81 (0.87)	3.03 (1.02)
Disto-Buccal	*T0*	3.65 (2.76)	3.29 (1.83)
	*T2*	5.55 (2.28) *	**7.13 (1.86)**
	*T3*	3.94 (1.67)	4.58 (1.73)
Disto-Lingual	*T0*	3.39 (2.20)	3.03 (1.47)
	*T2*	4.77 (1.61)	6.00 (1.91)
	*T3*	3.61 (1.43)	4.06 (1.84)

Abbreviations; T0, pre-operation; T2, 2 weeks post-operatively; T3, 14 weeks post-operatively

Results are shown as mean (SD)

^*^Indicates significant group difference (*P* < 0.05) between 2 groups

All radiographic scores (lamina dura, overall density, trabecular pattern) decreased at T1. During the healing period from T1 to T3, all radiographic scores for evaluation of bone regeneration increased in both groups with the collagen sponge insertion side being slightly higher. However, the difference was not significant (Table 4). The inter-examiner ICC was 0.83 (95% confidence intervals; 0.65–0.92) showing favorable agreement.

**Table 4:** Assessment of radiographic evaluation

Value	Time	Collagen sponge insertion	Control
**Lamina dura**	*T0*	0.10 (0.54)	0.13 (0.50)
	*T1*	−0.03 (0.31)	−0.06 (0.36)
	*T2*	0.10 (0.40)	0.00 (0.26)
	*T3*	0.32 (0.48)	0.16 (0.45)
**Overall Density**	*T0*	0.29 (0.82)	0.42 (0.62)
	*T1*	0.06 (0.44)	−0.03 (0.41)
	*T2*	0.48 (0.51)	0.29 (0.46)
	*T3*	1.16 (0.73)	0.84 (0.73)
**Trabecular pattern**	*T0*	0.52 (0.68)	0.35 (0.61)
	*T1*	0.29 (0.46)	0.13 (0.43)
	*T2*	0.39 (0.50)	0.29 (0.53)
	*T3*	0.71 (0.59)	0.61 (0.56)

Abbreviations; T0, Pre-operation; T1, 1 week post-operatively, T2, 2 weeks post-operatively; T3, 14 weeks post-operatively Results are shown as mean (SD)

## Discussion

Periodontal defects on distal to mandibular second molar was frequently reported after extraction of impacted mandibular third molar. In previous studies, it has been reported that 43.3% of 215 cases resulted in probing depths of 7 mm or greater 2 years after extraction of impacted mandibular third molar [17, 18]. Also, post-operative complications such as pain, swelling, trismus, healing, and bone regeneration disturbance developed after extraction of impacted mandibular third molar may affect the patient’s quality of life [19]. Various use of materials and methods have been introduced and investigated to obtain favorable results and reduce postoperative complications after the extraction of impacted mandibular third molar [11, 20, 21].

One of the various methods used is the insertion of collagen sponge after extraction of impacted mandibular third molar and it has been widely used in oral and maxillofacial surgery. Collagen sponge has long been used actively in the medical and dental field and there has been many studies proving its advantages. The benefits of such absorbable materials include promotion of wound healing, clot stabilization, wound stabilization, and hemostasis, enhancing primary wound coverage through its chemotactic ability to attract fibroblasts and augmenting flap thickness by providing collagenous scaffold [22].

Ateloplug® (Bio-land, Cheong-ju, Chungcheongbuk-do) collagen sponge was used in this study. The purpose of the study was to evaluate the effectiveness of the use of Ateloplug compared to no use based on its post-operative clinical complications, periodontal defects, healing, and radiographic bone healing. Split-mouth design was chosen to reduce bias coming from any single patient that may have affected the reliability of the study.

Mean VAS score was significantly lower in collagen sponge insertion side at 1 week post-operation than the control side (*P* < 0.05). In both groups, mean VAS scores gradually decreased during the follow up period. MMO was lowest 1 week post-operation then gradually increased. There was no difference in MMO between the experimental and control groups because this study was a split-mouth design. Facial swelling was lower in the collagen sponge insertion side in the early healing period of 1 and 2 weeks post-operation but became similar 14 weeks post-operation on both sides. However, there was no significant difference observed between the two groups during follow-up. This suggests that the insertion of collagen sponge after extraction of impacted mandibular third molar may reduce early stage post-operative complications. This beneficial effect is thought to be related to collagen sponge, which acts as an extracellular matrix and has the ability to enhance healing for extraction sockets by gradually absorbing and replacing new tissue and promote maturation. This process increases revascularization and fibroblastic activity that promote wound healing [23, 24].

There have been similar studies that examined healing trends after socket filling. As with our results, Cho et al. [25] reported relatively low incidence of surgical site infection, alveolar osteitis, and hematoma at extraction socket with type I collagen sponge insertion in their retrospective studies. Cortell et al. [26] reported that patients who received collagen membrane showed significant improvement of probing depth and bone fill and they also resulted in faster recovery compared to the control group. Additionally, platelet concentrates – platelet rich plasma [11] and platelet rich fibrin [27] - were found to reduce postoperative complications such as pain, swelling, osteitis, and trismus.

In this study, all patients received 7 days of antibiotics and analgesics but the necessity of antibiotic prescription in third molar extraction remains controversial. Gbotolorun et al. [28] reported that the prescription of antibiotics after routine intra-alveolar dental extraction in healthy patients may not play any significant role in preventing wound healing complications. Similarly, Arteagoitia et al. [29] mentioned that prophylactic use of amoxicillin does not significantly reduce the risk of infection and/or dry socket after third molar extraction in their systematic review and meta-analysis. On the other hand, there have been several systematic reviews advocating the use of oral systemic antibiotic therapy to minimize the risk of infection [30, 31]. Thus, the use of medication should be determined by the physician with careful consideration on individual benefits and risks.

In previous studies, it has been reported that 43.3% of cases resulted in PD of 7 mm or greater after extraction of impacted mandibular third molar at the distal site of mandibular second molar [18, 32, 33]. We took measurements at 4 points around the teeth— MB, B, DB, and DL sites. Measurement of mesio-lingual and lingual sites were excluded because the lingual flap elevation was uncommon during impacted mandibular third molar extraction and the change of PD was not considered to be significant. In our study, the baseline PD did not show any significant difference between two groups at all points. The mean PD was increased at T1 and gradually decreased throughout each follow-up at all points. During the survey period, the mean PD of collagen sponge insertion side was lower than that of the control side, especially at T1. There was a significant difference in PD between the two groups at DB site and the differences over varying time periods were also significantly different (*P* < 0.05). DB was the most affected site when full thickness muco-periosteal buccal flap elevation was done during the extraction of impacted mandibular third molar, suggesting that collagen sponge insertion into extraction socket may help the healing of periodontal defect and soft tissue in its early stages. This is based on the thought that collagen sponge maintains clusters of blood clot and promotes tissue healing and regeneration. Also, collagen sponge inhibits the collapse of soft tissue by maintaining the extracted socket space lacking alveolar bone support and prevents the inflow of food residues that can become the focus of infection [12]. The baseline of GR, GI, and BOP also did not show any significant difference between the experimental and control groups at all points. GI and BOP increased at 1 week post-operation in the two groups, which can be attributed to the possibility that surgery of impacted mandibular third molar extraction affected normal gingival conditions. GI and BOP decreased over time as gingival healing progressed in both groups, and the difference between the groups were not significant. GR both increased and decreased after surgery. Such seems to have been caused by the swelling of gingiva during the early post-operative period or by the loss of gingival adhesion during surgery.

After extraction, the socket is normally filled with blood clots and eventually the clots are replaced by granulation tissues. Granulation tissues are covered by connective tissue and osteoid develops from mesenchymal cells. Fibrillar coarse bone is formed and it is replaced by mature bone with osteoblastic and osteoclastic activity [34]. Collagen sponge has been used to help promote bone healing after extraction, but the effect of collagen sponge on promoting bone formation is debated. Several studies have reported that the porous structure of collagen sponge has advantages for colonization of seeded cells and that collagen sponge can increase bone formation by promoting osteoblast differentiation [35, 36]. However, Schoichoro Iwata et al. [37] reported significant difference of type I collagen mRNA between collagen sponge insertion and no insertion side, but no significant difference in the expression of osteocalcin mRNA, suggesting that collagen sponge did not accelerate cell proliferation or osteoinduction. In this study, collagen sponge insertion side scored higher radiographic evaluation than the control side during follow-up. However, the results should be carefully interpreted because the difference between the two groups was not statistically significant and the study was designed throughout a rather short study period of 14 weeks. Further study is warranted to evaluate the long-term prognosis and assessment of bone healing.

In accordance with the study protocol, all patients received same medications post treatment. However, individual adherence to medications may vary, thus the results should be carefully interpreted. To minimize bias, future studies should include a survey of individual adherence to each of the medications. Also, the current RCT did not cover various systemic diseases such as hypertension, diabetes, hypothyroidism, etc. as its exclusion criteria. Such lack of specificity may have lead to possible selection bias, thus future study setting should include all systemic diseases that may affect the healing of third molar extraction.

## Conclusion

Collagen sponge is a biocompatible material that fills the extraction socket and acts as a scaffold to prevent the collapse of soft tissue after extraction. In conclusion, it can help reduce the patient’s post-operative complications at an early stage and enhance initial healing of soft tissue and minimize periodontal defects.

## Supplementary information

**Additional file 1: Table S1.** Assessment of gingiva recession, gingival index and bleeding on probing.

## Data Availability

The datasets used and analyzed during the current study are available from the corresponding author on reasonable request.

## Abbreviations

HBV: Hepatitis B Virus
HIV: Human Immunodeficiency Virus
IV: Intravenous
MMO: Maximal mouth opening
PD: Probing Depth
VAS: Visual Analogue Scale
