# Supplementary material for: Randomized controlled trial on the effectiveness of absorbable collagen sponge after extraction of impacted mandibular third molar: split-mouth design
Source: BMC Oral Health. 2020 Mar 18;20:77. doi: 10.1186/s12903-020-1063-3 (PMC7079387; doi:10.1186/s12903-020-1063-3)
Supplement: Supplementary file 1 — Additional file 1: Table S1. Assessment of gingiva recession, gingival index and bleeding on probing. [file 12903_2020_1063_MOESM1_ESM.docx]

**Supplementary Table S1.** Assessment of gingiva recession, gingival index and bleeding on probing.

| Value | Time | Collagen sponge insertion | | Control |
| --- | --- | --- | --- | --- |
| **Gingiva recession** |  |  | |  |
| Mesio-Buccal | *T0* | 0.39 (0.76) | | 0.39 (0.76) |
|  | *T1* | 0.35 (0.66) | | 0.35 (0.66) |
|  | *T2* | 0.35 (0.66) | | 0.26 (0.44) |
|  | *T3* | 0.35 (0.71) | | 0.35 (0.71) |
| Buccal | *T0* | 0.58 (0.76) | | 0.58 (0.76) |
|  | *T1* | 0.48 (0.68) | | 0.23 (0.50) |
|  | *T2* | 0.42 (0.56) | | 0.29 (0.46) |
|  | *T3* | 0.55 (0.72) | | 0.55 (0.72) |
| Disto-Buccal | *T0* | 0.65 (0.75) | | 0.58 (0.76) |
|  | *T1* | 0.58 (0.72) | | 0.55 (0.89) |
|  | *T2* | 0.52 (0.63) | | 0.61 (0.84) |
|  | *T3* | 0.65 (0.75) | | 0.90 (0.91) |
| Disto-Lingual | *T0* | 0.58 (0.76) | 0.58 (0.76) | |
|  | *T1* | 0.42 (0.50) | 0.52 (0.57) | |
|  | *T2* | 0.42 (0.50) | 0.42 (0.50) | |
|  | *T3* | 0.61 (0.72) | 0.74 (0.73) | |
| **Gingiva index** |  |  |  | |
| Mesio-Buccal | *T0* | 0.19 (0.48) | 0.13 (0.43) | |
|  | *T1* | 0.45 (0.68) | 0.58 (0.76) | |
|  | *T2* | 0.39 (0.50) | 0.32 (0.54) | |
|  | *T3* | 0.19 (0.40) | 0.16 (0.37) | |
| Buccal | *T0* | 0.16 (0.37) | 0.13 (0.43) | |
|  | *T1* | 0.65 (0.71) | 0.81 (0.75) | |
|  | *T2* | 0.39 (0.50) | 0.52 (0.57) | |
|  | *T3* | 0.23 (0.43) | 0.23 (0.43) | |
| Disto-Buccal | *T0* | 0.26 (0.58) | 0.19 (0.48) | |
|  | *T1* | 1.06 (0.63) | 1.32 (0.65) | |
|  | *T2* | 0.68 (0.60) | 0.94 (0.51) | |
|  | *T3* | 0.19 (0.40) | 0.29 (0.46) | |
| Disto-Lingual | *T0* | 0.23 (0.50) | 0.19 (0.40) | |
|  | *T1* | 0.77 (0.67) | 0.77 (0.67) | |
|  | *T2* | 0.65 (0.61) | 0.61 (0.62) | |
|  | *T3* | 0.16 (0.37) | 0.23 (0.43) | |
| **Bleeding on probing** |  |  |  | |
| Mesio-Buccal | *T0* | 0.16 (0.37) | 0.13 (0.34) | |
|  | *T1* | 0.52 (0.51) | 0.58 (0.50) | |
|  | *T2* | 0.26 (0.44) | 0.32 (0.48) | |
|  | *T3* | 0.06 (0.25) | 0.03 (0.18) | |
| Buccal | *T0* | 0.16 (0.37) | 0.13 (0.43) | |
|  | *T1* | 0.65 (0.71) | 0.81 (0.75) | |
|  | *T2* | 0.39 (0.50) | 0.52 (0.57) | |
|  | *T3* | 0.23 (0.43) | 0.23 (0.43) | |
| Disto-Buccal | *T0* | 0.26 (0.58) | 0.19 (0.48) | |
|  | *T1* | 1.06 (0.63) | 1.32 (0.65) | |
|  | *T2* | 0.68 (0.60) | 0.94 (0.51) | |
|  | *T3* | 0.19 (0.40) | 0.29 (0.46) | |
| Disto-Lingual | *T0* | 0.23 (0.50) | 0.19 (0.40) | |
|  | *T1* | 0.77 (0.67) | 0.77 (0.67) | |
|  | *T2* | 0.65 (0.61) | 0.61 (0.62) | |
|  | *T3* | 0.16 (0.37) | 0.23 (0.43) | |

Abbreviations; T0, Pre-operation; T1, 1 week post-operatively; T2, 2 weeks post-operatively; T3, 14 weeks post-operatively. Results are shown as mean(SD). ^*^ Indicates significant group difference (*P*<0.05) between 2 groups.
